# Alignment-free methods for metagenomic profiling

**DOI:** 10.1186/1471-2105-16-S15-P4

**Published:** 2015-10-23

**Authors:** Shanshan Gao, Diem-Trang Pham, Vinhthuy Phan

**Affiliations:** 1 Department of Computer Science, University of Memphis, Memphis, TN 38152, USA

## Background

The primary goal of metagenomic studies is to analyze and evaluate the rich microbial communities present in all natural environments. The construction and utilization of a large index required by alignment-based methods for thousands of microbial genomes can be computationally prohibitive. To avoid this computational cost, we investigated three different variations of an alignment-free method for profiling abundances of microbial communities.

## Materials and methods

The main idea of the method is reformulate the problem of determining abundance of microbial genomes as finding optimal solutions of linear equations that satisfy specific constraints. A set of genomic markers for the entire set of genomes is represented by a matrix F, where **F_ij_** represents the frequency of marker **i** in genome **j**. The occurrence vector **b** represents the frequencies of markers in reads. We would like to find an optimal solution **x**, the abundance vector in which **x_j_** represents the abundance of genome **j**. To find the abundance vector x, we solve the linear equation **Fx = b**. The methods to choose F and b are the key factor to find the optimal value of x. We introduced a concept of *genome specific marker* (GSM), which is a kmer that occurs in only one genome and no other. We exhaustively determine such markers from the entire dataset and represent the frequencies of these markers in the matrix F. Given a set of reads from a metagenomic dataset, we compute the frequency of GSM as b. Then, three variations can be formulated, respectively, as a linear programming problem (LP), a least-square approximation problem (L2), and an L1-approximation problem.

## Results

So far, our investigation on two data sets consisting of 100 and 1105 microbial genomes showed that the linear programming formulation (LP) yielded the best prediction of abundances of microbial genomes. This result was consistent across different levels of abundances. The LP variant also achieved better results across the board compared to a popular metagenomic profiler, FOCUS[1], which was found to be superior to other methods.

## Conclusions

In the future, we need to investigate deeper into the matrix F which consists not only of the GSM, but also the kmers that occur in more than one genome.
